# Evidence-based pharmacological prophylaxis recommendations for venous thromboembolism in hospitalized acutely ill medical patients: a systematic review of clinical practice guidelines

**DOI:** 10.1590/1677-5449.202300672

**Published:** 2023-08-07

**Authors:** Ana Paula Callejo de Souza, Franciele Cordeiro Gabriel, Géssica Caroline Henrique Fontes-Mota, Mariana de Siqueira Silva, Eliane Ribeiro

**Affiliations:** 1 Universidade de São Paulo - USP, São Paulo, SP, Brasil.

**Keywords:** venous thromboembolism, anticoagulants, practice guideline, hospitals, patient care, systematic review, tromboembolia venosa, anticoagulantes, guia de prática clínica, hospitais, assistência ao paciente, revisão sistemática

## Abstract

Venous thromboembolism is a complex multifactorial disease considered the most common cause of preventable deaths in hospitalized patients. Recommendations about pharmacological venous thromboembolism prophylaxis in adult hospitalized patients are available in clinical practice guidelines for optimization of healthcare delivery and improvement of patient outcomes. We conducted a systematic review of clinical practice guidelines using ADAPTE to synthesize recommendations for pharmacological prophylaxis of venous thromboembolism in hospitalized medical patients at a medium complexity university hospital. Recommendations for pharmacological prophylaxis were extracted from seven clinical practice guidelines considered of high quality after assessment with the Appraisal of Guidelines for Research and Evaluation (AGREE II) instrument. These recommendations will support discussion with specialists and implementation of practices in the setting of the hospital studied.

## Resumo

O tromboembolismo venoso é uma doença multifatorial complexa, considerada uma causa comum de óbitos evitáveis em pacientes hospitalizados. Recomendações sobre profilaxia farmacológica de tromboembolismo venoso em pacientes adultos hospitalizados estão disponíveis em diretrizes clínicas para otimizar os cuidados à saúde e contribuir com a melhora do desfecho do paciente. Dessa forma, foi conduzida uma revisão sistemática de diretrizes clínicas utilizando a metodologia ADAPTE para sintetizar as recomendações para profilaxia farmacológica de tromboembolismo venoso em pacientes clínicos adultos hospitalizados em um hospital universitário de média complexidade. As recomendações para profilaxia farmacológica foram extraídas de sete diretrizes clínicas consideradas de alta qualidade após avaliação pelo *Appraisal of Guidelines for Research and Evaluation* (AGREE II). Essas recomendações servirão de apoio para discussão com especialistas e implementação de práticas dentro do contexto do hospital estudado.

## INTRODUCTION

Venous thromboembolism (VTE), a complex multifactorial disease, is considered the most common cause of preventable deaths in hospitalized patients and thromboprophylaxis is an important strategy to improve patient safety in hospitals.^1^ The annual average incidence of thrombotic events in the United States and Europe varies between 1 and 2 per 1000 in the adult population, depending on age, sex, race and medical conditions.^2^ The incidence of confirmed hospital-acquired deep vein thrombosis is 10 to 40% in medical and surgical patients in the absence of thromboprophylaxis.^3^ Prophylaxis for VTE is well-established in worldwide guidelines, but 37% of medical and surgical patients at moderate risk and 29% of those at high risk are not given adequate prophylaxis.^4^

Clinical Practice Guidelines (CPGs) are tools that contain recommendations on clinical health interventions based on systematic reviews of evidence and constitute an important element for improving VTE prevention.^5,6^ Organizations may choose to adopt recommendations from existing CPGs, develop CPGs with recommendations based on available evidence, or adapt existing recommendations extracted from CPGs, considering local context.^7^ Adaptation is an efficient option to avoid duplication of guidelines and ensure implementation of recommendations that consider the local cultural and organizational context.^8^ The adaptation process must be rigorous and transparent to produce a high-quality CPG.^9^

The ADAPTE Collaboration, an international collaboration of researchers, guideline developers, and guideline implementers, has developed a tool taking a systematic approach to adaptation of high-quality CPGs for health care institutions considering the organizational and cultural environment for application in a different context. Matrices of recommendations and evidence levels must be drawn from the CPGs and grouped together by similarity, helping specialists to identify recommendations with strong evidence and clinical relevance.^10^ Several organizations have used the ADAPTE framework for CPG adaptation.^11^

Given the epidemiological importance of the disease, and in order to promote safe practices for prophylaxis of venous thromboembolism, we conducted a systematic review of published CPGs to synthesize recommendations for pharmacological VTE prophylaxis of medical patients hospitalized in a medium complexity teaching hospital by similar meaning.

## METHOD

### Study design

A systematic review was conducted to identify high-quality CPGs to obtain a synthesis of their recommendations for pharmacological VTE prophylaxis of hospitalized adult medical patients. The study was registered on the protocol registration portal, International Prospective Register of Systematic Reviews (PROSPERO), under number CRD42021232578, and is written following the guidelines of the Preferred Reporting Items for Systematic Reviews and Meta-Analyses (PRISMA).^12^

### Clinical question

The first step was formulating the clinical question grounded on the acronym P (population), I (intervention), P (professional), O (outcome), H (health system), as described in Table 1.

**Table 1:** Description of the PIPOH acronym used to define the clinical question on pharmacological prophylaxis of venous thromboembolism in adult hospitalized patients.

**PIPOH**	**INCLUSION CRITERIA**
Population	Hospitalized (> 24 hours) adults (> 18 years)
Intervention	Pharmacological prophylaxis for venous thromboembolism
Professional	Multi-professional team at a hospital
Outcome	Prophylaxis of venous thromboembolism in hospitalized patients
Health system	Medium complexity teaching hospital that included urgency/emergency, medium complexity elective procedures, trauma care, and orthopedics^13^

Health question: Which drugs have clinical evidence for the prophylaxis of adult medical patients hospitalized in public institutions of medium complexity?

### Search strategy

The clinical question guided the search for CPGs through definition of descriptors and inclusion criteria. The inclusion criteria were: CPGs defined by the Institute of Medicine (IOM)^5^ open access, in up to date versions in English, Portuguese, or Spanish, published between January 1, 2011 and March 31, 2021.

The following items were outside the scope of this study: pregnant and postpartum women, pediatric patients, outpatients, patients being treated for VTE, and patients suspected of or diagnosed with COVID-19.

An electronic database search was conducted in April 2021 for CPGs on pharmacological prophylaxis in adult hospitalized patients. For all CPGs included in the study, the most up to date version available by December 2022 was sought.

A search strategy was implemented using the keywords “venous thromboembolism” and “guideline” on the following databases: Medical Literature Analysis and Retrieval System Online - Medline (via PubMed), the Cochrane Library (via CENTRAL), Embase, and Latin American & Caribbean Health Sciences Literature (LILACS). Search strings were constructed using specific indexing terms for each of the databases (Table 2).

**Table 2:** Search strategies used to obtain clinical practice guidelines with pharmacological prophylaxis for venous thromboembolism from PubMed, Cochrane Library, Embase, and Lilacs.

**DATABASES**	**SEARCH STRATEGY**
**MEDLINE (via PubMed)**	(“Practice Guidelines as Topic”(MeSH Terms) OR (“Practice Guidelines as Topic”(MeSH Terms) OR (“practice”(All Fields) AND “guidelines”(All Fields) AND “topic”(All Fields) OR “Practice Guidelines as Topic”(All Fields) OR (“clinical”(All Fields) AND “guidelines”(All Fields) AND “topic”(All Fields) OR (“Practice Guidelines as Topic”(MeSH Terms) OR (“practice”(All Fields) AND “guidelines”(All Fields) AND “topic”(All Fields) OR “Practice Guidelines as Topic”(All Fields) OR (“best”(All Fields) AND “practices”(All Fields) OR “best practices”(All Fields) OR (“Practice Guidelines as Topic”(MeSH Terms) OR (“practice”(All Fields) AND “guidelines”(All Fields) AND “topic”(All Fields) OR “Practice Guidelines as Topic”(All Fields) OR (“best”(All Fields) AND “practice”(All Fields) OR “best practice”(All Fields) OR (“Practice Guideline”(Publication Type) OR (“Practice Guideline”(Publication Type) OR “Practice Guidelines as Topic”(MeSH Terms) OR “clinical practice guideline”(All Fields) OR (“ambulatory care facilities”(MeSH Terms) OR (“ambulatory”(All Fields) AND “care”(All Fields) AND “facilities”(All Fields) OR “ambulatory care facilities”(All Fields) OR “clinic”(All Fields) OR “clinic s”(All Fields) OR “clinical”(All Fields) OR “clinically”(All Fields) OR “clinicals”(All Fields) OR “clinics”(All Fields) AND (“Guideline”(Publication Type) OR “guidelines as topic”(MeSH Terms) OR “guidelines”(All Fields) OR “Guideline”(Publication Type) AND (“Venous Thromboembolism”(MeSH Terms) OR (“Venous Thromboembolism”(MeSH Terms) OR (“venous”(All Fields) AND “thromboembolism”(All Fields) OR “Venous Thromboembolism”(All Fields) OR (“thromboembolism”(All Fields) AND “venous”(All Fields) OR “thromboembolism venous”(All Fields)
**Embase**	('practice guideline'/exp/mj OR 'clinical practice guidelines'/mj OR 'guidelines'/mj OR 'guidelines as topic'/mj OR 'practice guideline'/mj OR 'practice guidelines'/mj OR 'practice guidelines as topic'/mj) AND ('venous thromboembolism'/exp OR 'thromboembolism, venous' OR 'vein thromboembolism' OR 'venous thromboembolism') AND (2011-2021)/py AND (embase)/lim
**Cochrane**	#1 MeSH descriptor: (Venous Thromboembolism) explode all trees
	#2 (Thromboembolism, Venous)
	#3 #1 OR #2
	#4 MeSH descriptor: (Practice Guidelines as Topic) explode all trees
	#5 Clinical Guidelines as Topic) OR (Best Practices) OR (Best Practice) OR (Practice Guideline) OR (Clinical Guidelines) OR (Guideline)
	#6 #4 OR #5
	#7 #3 AND #6
**LILACS**	“thromboembolism” OR “venous thromboembolism” (words) AND (“guideline” OR “guideline/protocol” OR “guidelines as topic” OR “guidelines/consensus”) OR “guide of clinical practice” OR “guide of medical practice”) OR “guideline” OR “medical practice guideline” OR “clinical practice guideline” OR “medical practice guideline” OR “guideline/protocol” (words)

A manual search was also conducted on specific CPG repositories and organization websites using the keyword “venous thromboembolism”: Australian Clinical Practice Guidelines (clinicalguidelines.gov.au), Canadian Agency for Drugs and Technologies in Health (cadth.ca), International Guidelines Network (gin.net), ECRI Guidelines Trust (guidelines.ecri.org), Scottish Intercollegiate Guidelines Network (sign.ac.uk), Queensland Health (health.qld.gov.au), American Society of Hematology (hematology.org), American College of Physicians (acponline.org), American College of Chest Physicians (chestnet.org), International Union of Angiology (angiology.org), National Institute for Health and Care Excellence (nice.org.uk), National Guidelines Clearing House (guidelines.gov), European Society of Anaestheology and Intensive Care (esaic.org), and Thrombosis Canada (thrombosiscanada.ca)

### Selection of clinical practice guidelines

The references retrieved were exported to Rayyan ® reference manager and duplicates were excluded (duplicates not found by the software were deleted manually). Two reviewers (APCS and FCG) independently screened the retrieved titles and abstracts. After the first screening, two reviewers (APCS and FCG) screened the full texts.

Discrepancies found in this process were discussed between the two reviewers and resolved through consensus. When no consensus was reached, a third reviewer participated in the discussion. The same method was used for all subsequent processes until conclusion of extraction of the recommendations.

### Data extraction

Two reviewers (APCS and MSS) independently extracted the following data, from each of the included CPGs, using a piloted standard form on Google Forms®, followed by transfer to an Excel® spreadsheet: year of publication, development country, development institution or organization, development method, formal consensus for formulation of the recommendation, financial contributions, funding organizations, classification of evidence, application of GRADE, professionals involved in the development group, perspectives from the patient, patients in the development group, external review, and predicted update schedule.

### Quality assessment

The quality assessment was conducted by three reviewers (APCS, FCG & GCHF-M) independently trained in the AGREE II instrument. The training process included an initial discussion of the AGREE II manual, followed by assessment of the quality of CPGs for the treatment of chronic pain^14^ and Gaucher’s disease^15^ on the online platform My AGREE Plus. After these assessments, reviewers and trainer discussed the scores and the discrepancies. Finally, the team evaluated two recent CPGs for obesity and hyperthyroidism^16,17^ and engaged in a discussion about the tool and discrepancies.

The AGREE II instrument consists of 23 items grouped in six domains. Each item was given three grades, one from each reviewer, directly on the online platform My AGREE Plus.^18^ Each AGREE II item is scored using a 7-point Likert scale, where 1 indicates that there is no information for the AGREE II item and 7 indicates that the information is of the highest possible quality.^10^ Grades were considered discrepant when there was a difference of two or more points between different reviewers’ grades.

A high-quality CPG was defined using the AGREE II domain scores, with a cut-off of 60% or more for AGREE II domains 3 (rigor of development) and 6 (editorial independence).^19^

### Synthesis of recommendations

Two reviewers (APCS and MSS) independently read each CPG to acquire an overall impression of their content and procedures and extracted recommendations to an Excel® spreadsheet, using an exact translation of the wording of each recommendation. We compared the different therapeutic strategies and terminologies between CPGs and similar recommendations were synthesized in a table with the level of evidence and the strength of each recommendation.

### Ethics and dissemination

No ethical approval is required for this type of systematic review, since no patient data is used. The research results will be presented at conferences and submitted to a peer reviewed journal.

## RESULTS

### Selection of CPGs

**Figure 1:**
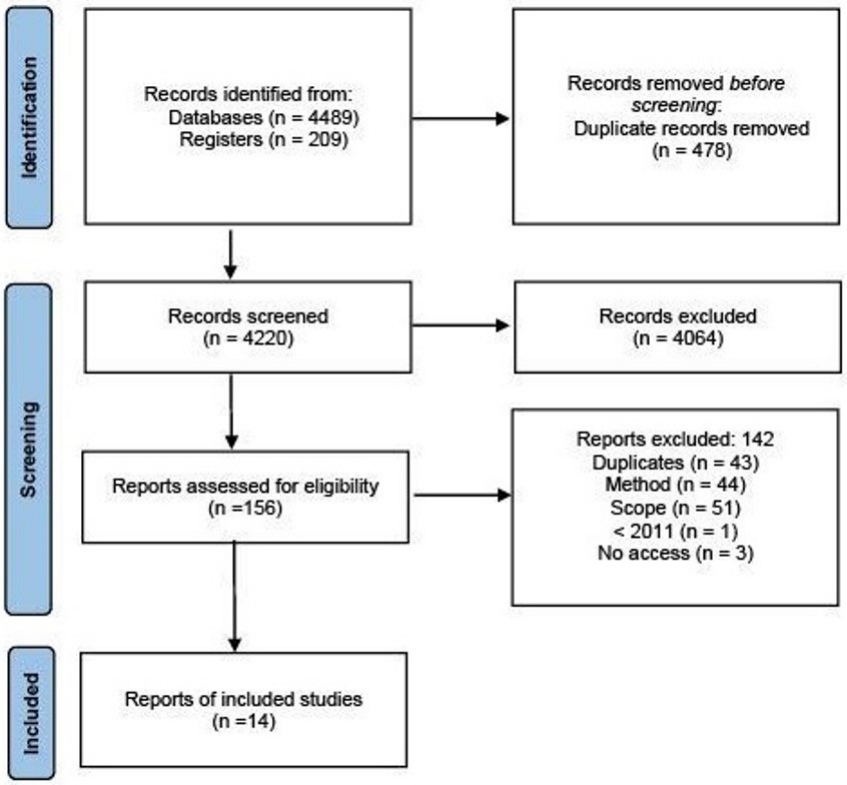
A flow chart summarizing the results of the literature search and selection of clinical practice guidelines.

The bibliographic search identified 4,698 records from databases of which 478 were duplicates. The authors screened the 4,220 remaining references by title and abstract and excluded 4,064 that were not in the selection criteria. They then reviewed the full text of 156 documents and excluded 142 documents. Fourteen CPGs were included (Figure 1).

### Data extraction

General characteristics of the fourteen (14) CPGs were extracted. In summary, three CPGs were published in 2011,^20-22^ two in 2012,^23,24^ three in 2013,^25-27^ one in 2014,^28^ one in 2015,^29^ one in 2016,^30^ and three in 2018.^31-33^ CPGs were developed in Argentina,^26^ Australia,^24,32^ England,^31^ Germany,^29^ Japan,^20^ Malaysia,^27^ Mexico,^21^ Saudi Arabia,^30^ Scotland,^28^ and the United States,^25^ and one was developed by an International Union.^22,23,33^ Just one was developed by a group of researchers,^26^ while six were developed by government organizations,^24,27,28,30-32^ and seven by professional societies.^20-23,25,29,33^

Most CPGs were developed by systematic review^21-23,25-33^ and only four used a formal consensus to formulate the recommendations.^21,29,30,33^ Three CPGs did not mention financial contributions or funding organizations.^20,21,26^ Most CPGs rated the quality of the evidence,^20,22-33^ but only six used the GRADE system.^22,23,28,30,31,33^ Five CPGs included a multi-professional team in the development group,^24,27,28,31,33^ nine included patients’ perspectives in the CPG,^23-25,28-33^ but eleven did not include patients in the development group.^20-27,29-31^ Finally, nine mentioned that the CPG was subjected to external review^22,23,25,27-31,33^ and only six informed a predicted updating schedule.^22,24,28,29,31,33^

### CPG quality assessment

The methodological quality of fourteen (14) CPGs was assessed using the AGREE II instrument and seven guidelines obtained scores greater than or equal to 60% in domains 3 and 6 and were thus defined as of high quality, as follows: 1. National Institute for Health and Care Excellence: Venous thromboembolism in over 16s Reducing the risk of hospital-acquired deep vein thrombosis or pulmonary embolism (NICE),^31^ 2. American Society of Hematology guidelines for management of venous thromboembolism: Prevention of venous thromboembolism in surgical and medical hospitalized patients (ASH),^33^ 3. Scottish Intercollegiate Guidelines Network: Prevention and management of venous thromboembolism (SIGN),^28^ 4. German interdisciplinary, evidence- and consensus-based: Clinical practice guideline: The prophylaxis of venous thromboembolism (AWMF),^29^ 5. National Health and Medical Research Council: Clinical practice guideline for the prevention of venous thromboembolism in patients admitted to Australian hospitals (NHRMC),^24^ 6. American College of Chest Physicians: Antithrombotic Therapy and Prevention of Thrombosis, 9th ed: American College of Chest Physicians Evidence-Based Clinical Practice Guidelines (ACCP),^23^ and 7. American College of Physicians: Venous thromboembolism prophylaxis in hospitalized patients: A clinical practice guideline from the American College of Physicians (ACP).^22^

### Synthesis of recommendations

The synthesis of the recommendations for pharmacological prophylaxis of VTE in acutely ill medical patients is provided in Table 3.

**Table 3:** Synthesis of recommendations for pharmacological prophylaxis indications and strategies in acutely ill medical patients extracted from high quality clinical practice guidelines published from January 2011 to March 2021.

**Recommendation**	**Clinical Practice Guideline**	**Level of Evidence**	**Strength of the recommendation**
After a risk assessment consider pharmacological prophylaxis with:	ACP	Moderate-quality evidence moderately confident in the effect estimate: the true effect is likely close to the estimated effect, but there is a sizable possibility that it is substantially different	Strong
*Low Molecular Weight Heparin*	ACCP	GRADE 1B	Strong
(ACCP, NHMRC, SIGN^a^, AWMF^b^, ASH^c^ and NICE^d^)		Randomized controlled trials with important limitations (inconsistent results, methodologic flaws, indirect or imprecise) or very strong evidence from observational studies	
*Unfractionated heparin*	NHRMC	Body of evidence can be trusted to guide practice in most situations	B
(ACP, ACCP, NHMRC, SIGN^a^; ASH^c^),	SIGN	At least one meta-analysis, systematic review, or randomized controlled trials rated as 1++ and directly applicable to the target population; or a body of evidence consisting principally of studies rated as 1+, directly applicable to the target population, and demonstrating overall consistency of result	A
*Fondaparinux*	AWMF	High quality of evidence (systematic review with or without meta-analysis or randomized controlled trials)	Strong
(ACCP, SIGN^a^, AWMF^b^, ASH^c^, NICE^d^)	ASH	Low certainty in the evidence of effects (certainty in these estimated effects was rated as very low owing to risk of bias and imprecision of the estimates)	Conditional (suggest)
	NICE	No specification^e^	Strong
For acutely ill hospitalized medical patients *at low risk of thrombosis*, the recommendation is not to use pharmacologic prophylaxis or mechanical prophylaxis based on the Padua Prediction Score	ACCP	GRADE 1B	Strong
		Randomized controlled trials with important limitations (inconsistent results, methodologic flaws, indirect or imprecise) or very strong evidence from observational studies	
For acutely ill hospitalized medical patients who are *bleeding or at high risk for bleeding*, the recommendation is not to use anticoagulant thromboprophylaxis based on the Padua Prediction Score	ACCP	GRADE 1B	Strong
		Randomized controlled trials with important limitations (inconsistent results, methodologic flaws, indirect or imprecise) or very strong evidence from observational studies	
*Aspirin* is not recommended as the sole pharmacological agent for venous thromboembolism prophylaxis in medical patients	SIGN	A body of evidence including studies rated as 2+, directly applicable to the target population and demonstrating overall consistency of results; or extrapolated evidence from studies rated as 2++	C
Medical patients hospitalized for acute illness are recommended *Low Molecular Weight Heparin* over Direct Oral Anticoagulants for venous thromboembolism prophylaxis	ASH	Moderate certainty in the evidence of effects (certainty in these estimated effects was moderate owing to imprecision of the estimates for the VTE outcomes)	Strong
Drug prophylaxis for patients should generally *last 6 to 14 days*.	ACCP^f^	GRADE 2B	Weak
		Evidence from randomized controlled trials with important limitations (inconsistent results, methodologic flaws, indirect or imprecise) or very strong evidence from observational studies.	
	AWMF	Moderate	Recommended
		Randomized controlled trials or cohort studies of limited quality	
	NICE^g^	No specification^e^	Strong
Recommends inpatient over inpatient plus extended-duration outpatient venous thromboembolism prophylaxis with heparin and direct oral anticoagulants	ASH	Moderate certainty in the evidence of effects certainty in these estimated effects was low owing to imprecision of the estimates and indirect comparisons)	Strong

a LMWHs are preferred to UFH because of their longer half-life, lesser tendency to cause heparin associated thrombocytopenia, and once daily dosing schedule (recommended best practice based on the clinical experience of the guideline development group)

b Preferably with LMWH in high-risk prophylaxis doses.

c LMWH (low certainty in the evidence of effects: certainty in these estimated effects was rated as very low owing to risk of bias and imprecision of the estimates) or fondaparinux (very low certainty in the evidence effects: certainty in these estimated effects was very low owing to the risk of bias, the indirect comparison, and imprecision of the estimates) rather than UFH.

d LMWH as first-line treatment and if LMWH is contraindicated use fondaparinux.

e The quality of evidence was assessed by outcomes using GRADE. After evaluation, the evidence was interpreted for development of recommendations considering the balance between benefits, harm, and cost of each intervention. Recommendations based on weak, conflicting, or absent evidence were developed based on expert opinion. Strong recommendations were described as “offer” and weak recommendations as “consider”.

f Until full mobility is restored or until discharge from hospital, whichever comes first.

g At least 7 days.

## DISCUSSION

In this review, we systematically identified seven (7) high quality CPGs and synthesized a list of recommendations from them, noting the level of evidence and the strength of the recommendations for pharmacological prophylaxis of VTE in acutely ill medical patients.

For pharmacological prophylaxis strategies in acutely ill medical patients, the CPGs recommended low molecular weight heparin (LMWH),^23,24,28,29,31,33^ unfractionated heparin (UFH),^22-24,28,33^ and fondaparinux.^23,28,29,31,33^ The LMWH was cited as first choice by NICE^31^ because of the clinically beneficial effects and was preferred by the AWMF^29^ due to the lower risk of thrombocytopenia. SIGN^28^ and ASH^33^ suggested LMWH over UFH, with low evidence level, because of its long half-life, its lower likelihood of causing heparin-associated thrombocytopenia, and its once daily dosing schedule. ASH^33^ recommended LMWH over direct oral anticoagulants (DOAC) with moderate evidence, due to the increased risk of bleeding using DOAC compared with LMWH. For duration of prophylaxis, three CPGs^23,29,31^ recommended from 6 to 14 days and both ACCP^23^ and ASH^33^ recommended not extending prophylaxis for outpatients.

All hospitalized patients should be assessed for risk of VTE and bleeding and pharmacological prophylaxis should be initiated for patients without contraindications.^34^ Acutely ill medical patients are exposed to risk factors for developing VTE due their acute medical illness and the prolonged immobility during the illness.^35^

Prophylaxis and duration of treatment in medical patients has always been controversial and has been widely discussed. The findings of reviews recommend LMWH, UFH, and Fondaparinux for pharmacological prophylaxis.^35^

The Medical Patients with Enoxaparin Trial (MEDENOX) was a prospective, double-blind, randomized, placebo-controlled study conducted from 1996 to 1998 that recruited 1,102 medical patients from 68 centers and 9 countries aged over 40 years and hospitalized for at least 3 days with acute illness who were given low molecular weight heparin (enoxaparin) for 6 - 14 days and had a lower incidence of VTE compared with the placebo study group (5.5% vs. 15.0%, p <0.001).^36^

The Prospective Evaluation of Dalteparin Efficacy for Prevention of VTE in Immobilized Patients Trial (PREVENT) was an international, multicenter, randomized, double-blind, placebo-controlled study, conducted from July 2001 to April 2002 with the objective of evaluating the efficacy and safety of dalteparin for the prevention of VTE. It randomized 3,706 medical patients hospitalized for at least 4 days for acute illness aged over 40 years to receive dalteparin or placebo for 14 days. The group that received dalteparin had a lower incidence of VTE, 45% (*P*=0.0015) than the placebo group.^37^

A placebo-controlled trial to determine the efficacy of fondaparinux was conducted by the ARTEMIS group from March 2002 to January 2003, recruiting 644 patients aged over 60 with medical illness who received fondaparinux for 14 days and had a lower incidence of VTE (5.6%) than a placebo group (10.5%) (95% CI 7.7% to 69.3%).^38^

Prolonged prophylaxis for acutely ill clinical patients has shown equal benefit for reducing VTE, but has also led to an increased risk of bleeding in acutely ill clinical patients.

The Extended Prophylaxis for Venous Thromboembolism in Acutely Ill Medical Patients With Prolonged Immobilization (EXCLAIM) study was a clinical, multicenter, prospective, randomized, double-blind, placebo-controlled trial of extended prophylaxis for thromboembolism in 4,726 patients that aimed to compare the efficacy and safety of extended prophylaxis of VTE with enoxaparin for a longer period of time (28 +/- 4 days) to placebo after enoxaparin prophylaxis for 10 +/- 4 days in both groups. In the extended enoxaparin prophylaxis group, the incidence of VTE was lower compared to the placebo group (2.5% vs. 4%; with an absolute risk difference favoring enoxaparin -1.53% [95.8%CI, -2.54% to -0.52%]) but the number of bleeding events was higher (0.8% vs. 0.3%; with an absolute risk difference favoring placebo, 0.51% [95%CI, .12% to 0.89%]).^39^

A double-blind, double-dummy, placebo-controlled study (ADOPT) conducted from 2007 to 2011 recruited 4,495 acutely ill clinical patients to receive either apixaban for 30 days or enoxaparin for 6 to 14 days resulted in 2.71% VTE-related deaths among patients receiving extended prophylaxis with apixaban (60/2.211) compared to 3.06% among patients receiving short-term prophylaxis with enoxaparin (70/2.284) (relative risk with apixaban, 0.87; 95%CI, 0.62 to 1.23; P=0.44). Bleeding events occurred in 0.47% of the apixaban extended prophylaxis group (15/3.184) and in 0.19% of patients receiving enoxaparin (relative risk with apixaban, 2.58; 95%CI, 1.02 to 7.27; P=0.04).^40^

Another multicenter, randomized, parallel group study, conducted from 2007 to 2010, compared safety and efficacy of rivaroxaban or enoxaparin for prevention of venous thromboembolism in hospitalized acutely ill medical patients (MAGELLAN) given subcutaneous enoxaparin 40 mg once a day for 10±4 days and oral placebo for 35±4 days or subcutaneous placebo for 10±4 days and oral rivaroxaban 10 mg once a day for 35±4 days. It was concluded that rivaroxaban was non-inferior to enoxaparin for standard duration thromboprophylaxis and in extended use rivaroxaban reduced the risk of venous thromboembolism, but was associated with an increased risk of bleeding.^41^

These recommendations should be used by healthcare organizations to develop and implement contextualized information for health professionals through a critical assessment by experts of those synthesized in matrices. Issues such as developer and patient values and costs should also be considered.

The process of synthesizing recommendations for pharmacological prophylaxis of VTE in acutely ill medical patients was conducted in a systematic and transparent manner. Since the recommendations obtained reflect the content of the CPGs, the methodological quality appraisal conducted using AGREE, a tool accepted as a gold standard for guideline evaluation, was an important element. The recommendations were classified according to their evidence levels and recommendation grades. All steps were conducted by at least two reviewers.

However, the evaluation process is subjective and only the grades for 2 AGREE domains were used to define high quality CPGs. The AGREE is a methodological quality assessment tool and the recommendations of clinical practice guidelines that were excluded due to lack of detail in the description of their methods cannot be considered to have no credibility.

CPGs published in languages other than English, Spanish, or Portuguese were excluded. In addition, each CPG described their recommendations according to the local context, which makes it necessary to interpret recommendations to avoid changing their meaning.

## CONCLUSION

In conclusion, the present systematic review synthesized recommendations for pharmacological prophylaxis of VTE in acutely ill medical patients from seven high quality CPGs.

The clinical practice guidelines selected used different clinical questions, scoring systems, and consensus processes to indicate levels of evidence, which may explain discrepancies in the strength of their recommendations. They also considered the availability of studies, drugs approved by local regulatory agencies, and the local context, which may explain the differences between their medication preferences.

These evidence-based recommendations provide support for discussions with specialists to implement contextualized information in health professionals’ settings.
